# Dermoscopic evaluation of superficial spreading melanoma^☆^^☆☆^

**DOI:** 10.1016/j.abd.2020.06.012

**Published:** 2021-02-01

**Authors:** Fernanda Marques Trindade, Maria Luiza Pires de Freitas, Flávia Vasques Bittencourt

**Affiliations:** a Dermatology Service, Hospital da Polícia Militar, Belo Horizonte, MG, Brazil; b Dermatology Service, Hospital das Clínicas, Universidade Federal de Minas Gerais, Belo Horizonte, MG, Brazil

**Keywords:** Dermoscopy, Melanoma, Skin neoplasms

## Abstract

**Background:**

Dermoscopy increases the sensitivity of the diagnosis of melanoma, leading to its early identification and increasing the chances of cure.

**Objective:**

To describe the clinical and dermoscopic characteristics of superficial spreading melanomas, and to detect the differences between *in situ* and invasive 1-mm thick melanomas.

**Methods:**

This was a cross-sectional study in which dermoscopic images of 58 melanomas, grouped according to their thickness, were evaluated.

**Results:**

24 *in situ* melanomas were evaluated, 28 invasive melanomas with Breslow ≤ 1 mm (0.50 ± 0.22 mm) and six with Breslow > 1 mm (2.35 ± 2.02 mm). *In situ* melanomas were smaller than invasive melanomas. The most commonly found dermoscopic criteria were asymmetry (84.5%), three or more colors (81.0%), and atypical network (79.3%). A non-specific pattern was more common in *in situ* melanomas (p = 0.028) and atypical network in invasive melanomas with Breslow 1 mm presented inverted network (p = 0.018).

**Study limitations:**

The sample was selected by convenience, since it was necessary to have a preoperative photo of the tumor, which may have led to the loss of clinically less significant lesions, as well as those highly suggestive of melanoma.

**Conclusions:**

Melanomas in early stages showed a more frequent nonspecific pattern and atypical network, while invasive melanomas showed a multicomponent pattern, three or more colors, and an inverted network.

## Introduction

Melanoma accounts for only 5% of skin tumors, but it is responsible for approximately 80% of deaths, due to its high potential for metastasis.^1^ Its incidence has been increasing worldwide in recent decades; however, a tendency to stabilization of the mortality rate has also been observed,^2^ probably due to the increase in the diagnosis of initial melanomas.

The most important prognostic factor is the thickness, or Breslow index.^2^, ^3^ The current system of the American Joint Committee on Cancer (AJCC) uses the Breslow index for staging, selecting the 1-mm-thick cutoff point for the definition of thin melanomas.^4^ The prognosis is directly related to early diagnosis, since the ten-year survival rate in thin melanomas is 93%.^4^, ^5^ In turn, survival is inversely proportional to the thickness of the tumor; melanomas with Breslow index > 4 mm show a ten-year survival of only 39%.^4^

Melanomas are classified into four main subtypes, which differ in terms of epidemiology, clinical presentation, dermoscopy, histopathology, molecular aspects, and evolution, and therefore must be studied separately. Superficial spreading melanoma (SSM) is the most common subtype in fair-skinned individuals, corresponding to 70% of cases.^2^ It presents a radial growth phase, defined by growth limited to the epidermis or focused on the papillary dermis, followed by a phase of vertical growth with the presence of invasion.^2^

Dermoscopy is a non-invasive propaedeutic method that increases the accuracy of the diagnosis of melanoma by 20% to 30% when used by trained examiners, and its routine use by the dermatologist in the evaluation of melanocytic lesions is advisable.^6^, ^7^, ^8^ Follow-up in centers specialized in dermoscopy leads to the diagnosis of thinner melanomas, in addition to reducing the number of unnecessary biopsies.^9^, ^7^, ^10^, ^11^

The thinner the melanoma, the greater the difficulty in dermoscopic evaluation, due to a lesser structural breakdown present in the initial lesions.^12^, ^13^, ^14^ Several studies have been conducted in order to identify the dermoscopic criteria associated with the thickness of the tumor, aiming to obtain a preoperative estimate of the Breslow index, as well as to assist in the early diagnosis of melanoma, but the results are variable and none of these criteria proved to be reliable to establish the surgical approach.^15^, ^16^, ^17^, ^18^ Due to the importance of early diagnosis of melanoma, this study assessed the dermoscopic images of these tumors in order to describe and analyze the clinical and dermoscopic aspects of SSM, correlating them with the thickness of the tumor, which was stratified as in situ, invasive with Breslow index ≤ 1 mm, and invasive with Breslow index > 1 mm.

## Methods

This was is a cross-sectional study which included patients with melanomas diagnosed at the Pigmented Lesions Outpatient Clinic of the Dermatology Service of the Hospital das Clínicas (HC) of the Universidade Federal de Minas Gerais (UFMG) and in a private clinic over a period of seven years with a diagnosis of SSM confirmed by histopathology, who had a dermoscopic photographic record of the tumor.^19^ Data were collected through the image bank and services database, in addition to analysis of medical records.

According to the routine of the services, patients were examined with Heine 20® or DermLite DL3® (non-polarized light) dermoscopes using gel as interface fluid, and clinical and dermoscopic photographs of the suspected lesions were obtained with Canon Power Shot 3.2® or Nikon 1® digital cameras; patients signed an informed consent form and suspected melanoma lesions were referred for surgical excision.

The diagnosis of the SSM was based on an anatomopathological examination performed by experienced dermatopathologists. In cases of doubt regarding the histological subtype, the slide was reassessed. The selected patients met the following inclusion criteria: primary melanoma classified histologically as SSM and with a satisfactory quality dermoscopic photographic record. The exclusion criteria were as follows: other melanoma subtypes, recurrent melanomas, and unsatisfactory photographic record.

The clinical aspects evaluated were tumor size (< 6 mm or ≥ 6 mm) and location (face, cervical, trunk, upper limbs, or lower limbs). The histopathological aspect evaluated was the Breslow index; the tumors were classified as invasive SSM with Breslow index ≤ 1 mm, invasive SSM with Breslow index > 1 mm, as well as in situ melanoma (ISM), in which the Breslow index is not applied.

The analysis of the images was performed blindly, without knowledge of the Breslow index and other variables, by two observers (FVB and MLPF) with experience in the area of dermoscopy who, together, agreed as to the presence or absence of the evaluated criteria.

The registered dermoscopic criteria and patterns are described in Table 1, Table 2, respectively.^20^, ^21^, ^22^, ^23^, ^24^, ^25^, ^26^, ^27^, ^28^ As many images were not examined with a polarized light dermoscope, chrysalises were not included in the study as they can only be seen with this type of device.^22^, ^23^

**Table 1:** Definition of the dermoscopic criteria analyzed in superficial spreading melanomas.

Criterion	Definition
Contour, colors, and structure asymmetry	Inequality when the injury is divided into 0, 1, or 2 perpendicular axes.^20^
Atypical pigmentary network	Irregular network with variation in color, thickness, and spacing of lines and holes, distributed asymmetrically.^20^
Atypical dots and globules	Rounded or oval structures, with variation in color, size and shape; irregularly distributed in the lesion.^20^, ^21^
Radial streaks and/or pseudopods	Linear structures or bulbous projections found at the edge of the lesion, which may be connected to the body of the tumor or to a pigmented network, brown to black in color.^20^, ^21^
Whitish-blue veil	Irregular area, without structures, with “ground glass” whitish-blue color, which covers part of the lesion. Pigmentation cannot occupy the entire extent of the lesion.^20^
Regression structures (white scar area and/or peppering)	Depigmented scar area with lighter coloration than normal skin and/or blue-grayish granules, described as a pepper-type pattern.^20^
Hypopigmented area without structure	Hypopigmented area without peripheral structures occupying more than 10% of the lesion.^20^
Blotch	Black, brown, or gray area, without structures.^20^
	Regular: blotch in the center of the lesion, surrounded by a pigmented network.^21^
	Irregular: more than one blotch, or a blotch with peripheral location.^21^
Atypical vases	Dotted, irregular linear, or polymorphic vessels.^20^
Inverted network	Hypopigmented network in which the light areas form the beams and the pigmented areas fill the holes.^21^, ^22^, ^23^, ^24^
Milky-red area	Globule and/or larger area of a milky-red color, blurred or unfocused, which corresponds to an elevated part of the lesion.^25^ It consists of a vascular pattern, without specific distinguishable vessels.^21^
Dermoscopic island	Circumscribed area with uniform pattern that differs from the rest of the lesion.^26^
Reticular bluish-gray area	Coarse bluish-gray net, with thick lines and large holes.^27^, ^28^
Presence of multiple colors	Presence of three or more colors, including black, light brown, dark brown, blue, red, white, and gray.

**Table 2:** Definition of the dermoscopic patterns analyzed in superficial spreading melanomas.

Pattern	Definition
Multicomponent pattern	Presence of three or more criteria.^20^
Non-specific pattern	Pigmented lesion without a specific pattern.^20^
Reticular pattern	Pigmented network covering most of the lesion.^20^
Globular pattern	Numerous globules of varying sizes with brown and/or blackish-gray tones.^20^
Starburst pattern	Radial streaks and/or pseudopods in radial arrangement at the edge of the lesion.^20^
Homogeneous pattern	Diffuse brown, blue or grayish-black pigmentation in the absence of other local structures.^20^

The statistical analysis was performed during the descriptive part of this study by calculating means and proportions of continuous and qualitative variables, respectively. Univariate analysis for comparison between groups was performed using the chi-squared test or Fisher's exact test. The software used was Stata v. 13. The qualification of statistical significance was done using the p-value limit of 0.05.

The project was approved by the Chamber of the Department of Clinical Medicine at the Faculdade de Medicina of UFMG and by the UFMG Research Ethics Committee (opinion No. ETIC - 0005.0.203.000-09).

## Results

A total of 76 SSM were selected in 62 patients, of which 18 were excluded due to poor quality of the photos. Therefore, 58 SSM were evaluated in 52 patients, 31 from the Pigmented Lesions Outpatient Clinic of HC – UFMG and 21 from the private clinic; 24 were classified as ISM, 28 as invasive SSM with Breslow ≤ 1 mm, and six as invasive SSM with Breslow > 1 mm.

Among the 34 invasive SSM, the mean Breslow index was 0.82 ± 1.08. In the invasive SSM group with Breslow ≤ 1 mm, the mean was 0.50 ± 0.22; while in the invasive SSM group with Breslow > 1 mm, it was 2.35 ± 2.02. The clinical aspects are shown in Table 3.

**Table 3:** Clinical aspects of superficial spreading melanomas.

Clinical aspects	In situ	≤ 1 mm	> 1 mm	Total	Comparisons (p-values)		
					General between 3 groups	In situ *vs*. invasive	≤ 1 mm *vs*. > 1 mm
Tumor size (n)	14	18	6	38			
< 6 mm	5 (35.7%)	0 (0%)	0 (0%)	5 (13.2%)	0.009	0.004	–
≥ 6 mm	9 (64.3%)	18 (100%)	6 (100%)	33 (86.8%)			
Location (n)	24	28	6	58			
Face	0 (0%)	0 (0%)	2 (33,3%)	2 (3.5%)	0.120	0.568	0.083
Cervical	1 (4.2%)	2 (7.1%)	0 (0%)	3 (5.2%)			
Trunk	16 (66.7%)	16 (57.1%)	4 (66.7%)	36 (62.1%)			
Upper limbs	1 (4.2%)	4 (14.3%)	0 (0%)	5 (8.6%)			
Lower limbs	6 (25.0%)	6 (21.4%)	0 (0%)	12 (20.7%)			

There was a loss of data in the size variable, since the study was retrospective, and in most cases the size of the lesion was not recorded in the medical records; this data was available in only 38 cases, and was classified as < or ≥ 6 mm. Only five (13.2%) measured < 6 mm, all of them in the ISM group, while 33 (86.8%) were ≥ 6 mm. ISM sizes were significantly smaller than invasive SSM (p = 0.004).

The trunk was the most frequent location, accounting for 62.1% of all tumors, with no difference between groups; in turn, 20.7% of melanomas were located on the lower limbs, 8.6% on the upper limbs, 5.2% on the cervical region, and 3.5% on the face (Table 3).

Table 4 presents the frequency of dermoscopic criteria and the comparisons between groups. The most frequently found dermoscopic characteristics were asymmetry (84.5%), three or more colors (81.0%), and atypical network (79.3%).

**Table 4:** Dermoscopic criteria identified in superficial spreading melanomas.

Dermatoscopic criteria	In situ	≤ 1 mm	> 1 mm	Total	Comparisons (p)		
					General	In situ *vs*. invasive	≤ 1 mm *vs*. > 1 mm
Asymmetry (n)	24	28	6	58			
No	3 (12.5%)	5 (17.9%)	1 (16.7%)	9 (15.5%)	0.877	0.722	1.000
Yes	21 (87.5%)	23 (82.1%)	5 (83.3%)	49 (84.5%)			
Atypical network (n)	24	28	6	58			
No	7 (29.2%)	2 (7.1%)	3 (50.0%)	12 (20.7%)	0.020	0.181	0.029
Yes	17 (70.8%)	26 (92.9%)	3 (50.0%)	46 (79.3%)			
Atypical dots/globules (n)	24	28	6	58			
No	10 (41.7%)	10 (35.7%)	3 (50.0%)	23 (39.7%)	0.783	0.792	0.653
Yes	15 (58.3%)	18 (64.3%)	3 (50.0%)	36 (60.3%)			
Streaks/pseudopods (n)	24	28	6	58			
No	16 (66.7%)	18 (64.3%)	5 (83.3%)	39 (67.2%)	0.548	0.938	0.638
Yes	8 (33.3%)	10 (35.7%)	1 (16.7%)	19 (32.8%)			
Veil (n)	24	28	6	58			
No	18 (75.0%)	14 (50.0%)	3 (50.0%)	35 (60.3%)	0.163	0.055	1.000
Yes	6 (25.0%)	14 (50.0%)	3 (50.0%)	23 (39.7%)			
Regression structures (n)	24	28	6	58			
No	20 (83.3%)	23 (82.1%)	5 (83.3%)	48 (82.8%)	0.993	0.922	1.000
Yes	4 (16.7%)	5 (17.9%)	1 (16.7%)	10 (17.2%)			
Hypopigmented area (n)	24	28	6	58			
No	21 (87.5%)	24 (85.7%)	5 (83.3%)	50 (86.2%)	1.000	1.000	1.000
Yes	3 (10.5%)	4 (14.3%)	1 (16.7%)	8 (13.8%)			
Blotchs (n)	24	28	6	58			
No	20 (83.3%)	24 (85.7%)	5 (83.3%)	49 (84.5%)	0.938	0.675	1.000
Yes	4 (16.7%)	4 (14.3%)	1 (16.7%)	9 (15.5%)			
Atypical vessels (n)	24	28	6	58			
No	19 (79.2%)	21 (75.0%)	5 (83.3%)	45 (77.6%)	0.904	0.808	1.000
Yes	5 (20.8%)	7 (25.0%)	1 (16.7%)	13 (22.4%)			
Inverted network (n)	24	28	6	58			
No	22 (91.7%)	24 (85.7%)	2 (33,3%)	48 (82.8%)	0.009	0.131	0.018
Yes	2 (8.3%)	4 (14.3%)	4 (66,7%)	10 (17.2%)			
Milky red area (n)	24	28	6	58			
No	16 (66.7%)	16 (57.1%)	5 (83.3%)	37 (63.8%)	0.491	0.702	0.370
Yes	8 (33.3%)	12 (42.9%)	1 (16.7%)	21 (36.2%)			
Dermoscopic island (n)	24	28	6	58			
No	22 (91.7%)	27 (96.4%)	6 (100%)	55 (94.8%)	0.706	0.564	1.000
Yes	2 (8.3%)	1 (3.6%)	0 (0%)	3 (5.2%)			
Reticular bluish-gray area (n)	24	28	6	58			
No	24 (100%)	25 (89.3%)	6 (100%)	55 (94.8%)	0.325	0.260	1.000
Yes	0 (0%)	3 (10.7%)	0 (0%)	3 (5.2%)			
3 or + colors (n)	24	28	6	58			
No	8 (33.3%)	3 (10.7%)	0 (0%)	11 (19.0%)	0,078	0.019	1.000
Yes	16 (66.7%)	25 (89.3%)	6 (100%)	47 (81.0%)			

Atypical network was found in 17 (70.8%) ISM, in 26 (92.9%) invasive SSM with Breslow < 1 mm, and in three (50.0%) invasive SSM with Breslow > 1 mm. Its presence was significantly lower in invasive SSM with Breslow > 1 mm when compared with invasive SSM with Breslow ≤ 1 mm (p = 0.029).

A whitish-blue veil was less common in the ISM group (six cases; 25.0%) when compared with the invasive SSM groups with Breslow ≤ 1 mm (14 cases; 50.0%) and invasive SSM with Breslow > 1 mm (three cases; 50.0%), but not enough to achieve statistical significance (p = 0.055).

The inverted network was more frequent in invasive SSM with Breslow > 1 mm, in which the four cases represented, proportionally, 66.7% of the sample, while the ISM group presented two cases (8.3%) and the invasive SSM group with Breslow ≤ 1 mm, four cases (14.3%). The fact that this characteristic is more common in the Breslow > 1 mm group caused both the general comparison between the three groups (p = 0.009) and the comparison between SSM with Breslow ≤ and > 1 mm (p = 0.018) to be statistically significant.

The presence of three or more colors, common to 47 tumors (81.0% of the total), was proportionally more frequent in the invasive SSM group with Breslow > 1 mm, with 100% (6 cases), *vs.* 89.3% (25 cases) in the ≤ 1 mm group and 66.7% (16 cases) in the ISM group. The difference was statistically significant when comparing ISM and invasive SSM (p = 0.019).

Asymmetry, atypical dots and globules, radial streaks and/or pseudopods, regression structures, hypopigmented areas, blotches, atypical vessels, milky red areas, dermoscopic islands, and reticular bluish-gray areas did not show significant differences in the proportions found among the groups.

Table 5 shows the dermoscopic patterns and the comparisons between groups.

**Table 5:** Dermoscopic patterns identified in superficial spreading melanomas.

Dermoscopic patterns	In situ	≤ 1 mm	> 1 mm	Total	Comparisons (p)		
					General	In situ *vs*. invasive	≤ 1 mm *vs*. > 1 mm
Multicomponent (n)	24	28	6	58			
No	16 (66.7%)	9 (32.1%)	3 (50.0%)	28 (48.3%)	0.062	0.026	0.641
Yes	8 (33.3%)	19 (67.9%)	3 (50.0%)	30 (51.7%)			
Non-specific (n)	24	28	6	58			
No	19 (79.2%)	27 (96.4%)	6 (100%)	52 (89.7%)	0.136	0.028	1.000
Yes	5 (20.8%)	1 (3.6%)	0 (0%)	6 (10.3%)			
Reticular (n)	24	28	6	58			
No	19 (79.2%)	21 (75.0%)	5 (83.3%)	45 (77.6%)	0.904	0.808	1.000
Yes	5 (20.8%)	7 (25.0%)	1 (16.7%)	13 (22.4%)			
Globular (n)	24	28	6	58			
No	22 (91.7%)	27 (96.4%)	5 (83.3%)	54 (93.1%)	0.307	1.000	0.362
Yes	2 (8.3%)	1 (3.6%)	1 (16.7%)	4 (6.9%)			
Starburst (n)	24	28	6	58			
No	21 (87.5%)	28 (100%)	5 (83.3%)	54 (93.1%)	0.103	0.297	0.176
Yes	3 (12.5%)	0 (0%)	1 (16.7%)	4 (6.9%)			
Homogeneous (n)	24	28	6	58			
No	23 (95.8%)	28 (100%)	6 (100%)	57 (98.3%)	0.517	0.414	–
Yes	1 (4.2%)	0 (0%)	0 (0%)	1 (1.7%)			

**Figure 1:**
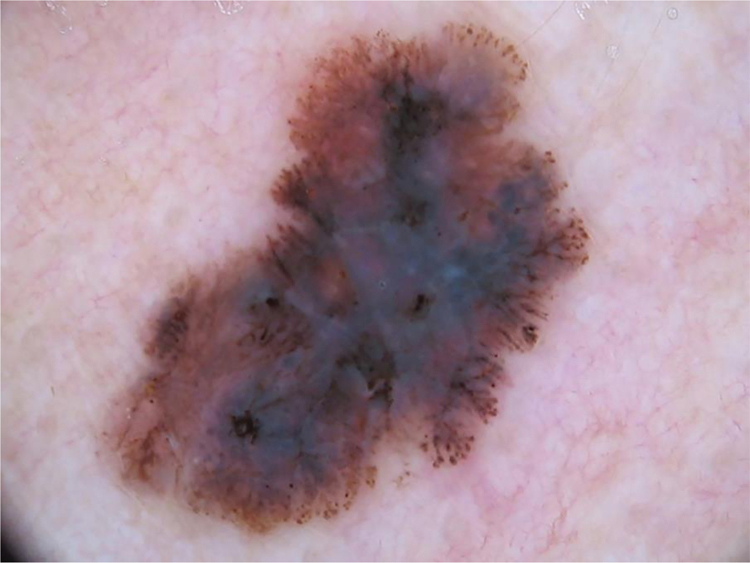
Superficial spreading melanoma with Breslow 0.5 mm located on the cervical region. Dermoscopy shows asymmetry of contour, colors and structures, atypical network, atypical dots and globules, radial streaks and pseudopods, and whitish-blue veil, featuring a multicomponent pattern.

**Figure 2:**
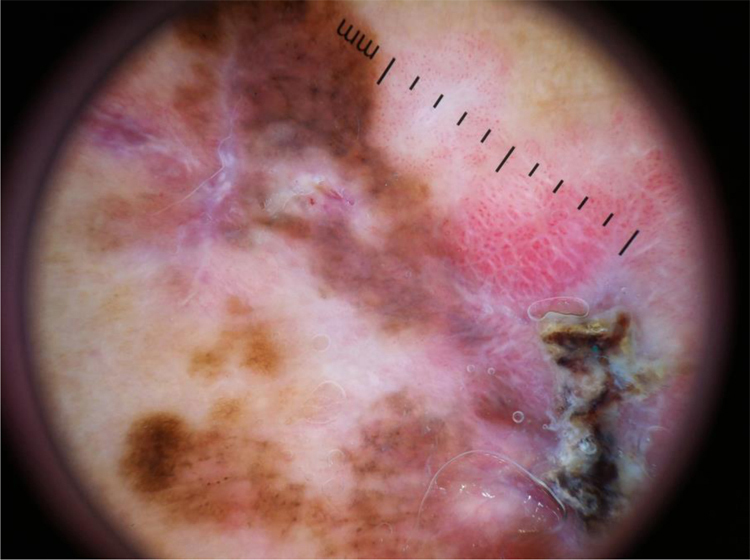
Superficial spreading melanoma with Breslow 0.6 mm located on the leg. Dermoscopy shows asymmetry, atypical network, atypical dots and globules, regression structures, atypical vessels, and a milky-red area, featuring a multicomponent pattern.

The multicomponent pattern (Figure 1, Figure 2) was observed in 30 (51.7%) tumors. Eight cases (34.8%) were observed in the ISM group, 19 (67.9%) and three (50.0%) cases in the invasive SSM groups with Breslow ≤ 1 mm and > 1 mm, respectively, being significantly more common in invasive SSM than in ISM (p = 0.026).

**Figure 3:**
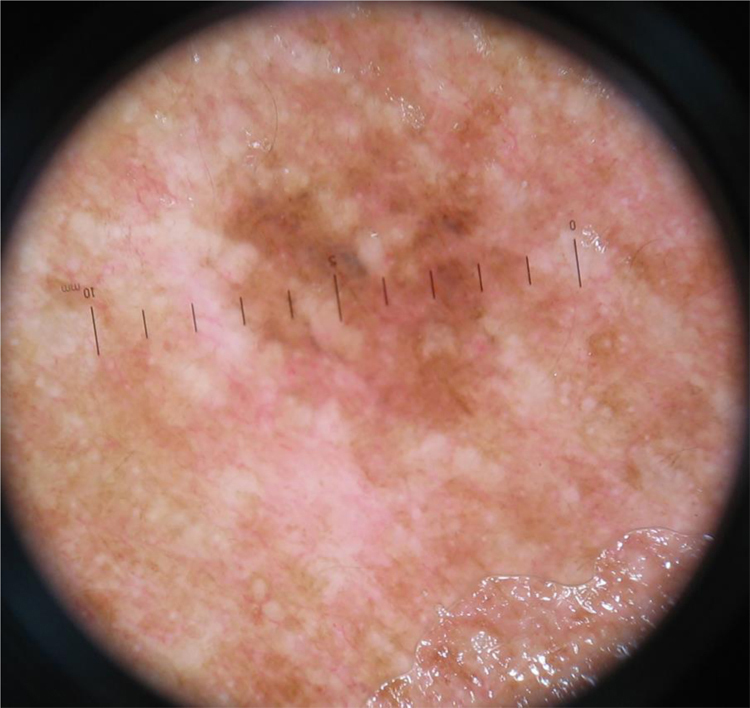
Superficial spreading melanoma in situ located on the cervical region, in a patient with multiple melanomas. Dermoscopy reveals asymmetry and color variation, however, without specific structures, characterizing a nonspecific pattern.

The nonspecific pattern (Fig. 3), common to six (10.3%) of the tumors, was more frequent in the ISM group (five tumors; 20.8%), when compared to the invasive SSM groups with Breslow ≤ 1 mm (one case; 3.6%) and > 1 mm (zero cases; 0%). The difference when comparing ISM with invasive SSM was statistically significant (p = 0.028).

The reticular, globular, homogeneous, and starburst patterns did not differ between groups.

## Discussion

The incidence of melanoma has increased alarmingly in recent decades worldwide, although with a tendency towards stabilization of the mortality rate.^29^ In fact, the world literature reports an increase in the incidence of thin melanomas, reflecting the earlier diagnosis, probably due to the use of dermoscopy. Another hypothesis would be that the improvement in the diagnosis of melanoma results in the detection of a subgroup of thin and slow-growing melanomas, which could progress only eventually to thick melanomas.^30^

The sample of 58 melanomas was obtained for convenience, since to be included in the study, the patient needed to have a pre-operative dermoscopic photo, which led to loss of cases, as clinically insignificant lesions may not have been photographed. In turn, patients with lesions that were clinically very suggestive of melanoma may have been referred to dermatology after tumor removal, leading to a loss of melanomas in advanced stages. The authors believe this was the reason for the group of invasive SSM with Breslow > 1 mm to have only six cases, making the average thickness of the invasive SSM only 0.82 mm. The sample can be considered expressive when compared with Brazilian studies.^15^, ^31^

The ISM had a mean size smaller than invasive melanomas, with a statistically significant difference (p = 0.04). This finding is expected and is in accordance with the literature, since smaller tumors tend to be less invasive.^32^

The dermoscopic characteristics most frequently found in this study were asymmetry, three or more colors, atypical network, and atypical dots and globules.

Asymmetry was the most common criterion (84.5%), with a similar frequency between groups, which is in accordance with previous studies.^15^, ^18^, ^32^ Asymmetry is a more frequent finding in SSM when compared with other subtypes of melanoma, and may be absent in nodular and amelanotic tumors, not included in this study.^33^, ^34^

The presence of three or more colors was significantly more common in invasive SSM than in ISM (p = 0.019), which is in accordance with the literature; it is suggested that the increase in the number of colors may be directly related to the Breslow index.^15^, ^18^, ^35^

In the phase of radial growth of melanoma, there is a proliferation of atypical melanocytes isolated or grouped in nests along the dermo-epidermal junction and in the suprabasal layers (pagetoid spread). This proliferation can cause changes in the pigmentary network, which becomes atypical, being a characteristic of melanomas of the SSM subtype, and may also be present in atypical nevi. Changes in the pigmentary network are more easily observed in early stage melanomas (ISM and invasive SSM with Breslow ≤ 0.75 mm), since with the progression of the lesion there is loss of epithelial ridges, and the network can be seen only focally.^36^, ^37^, ^38^, ^39^, ^40^ In this study, the atypical network was more common in invasive SSM with Breslow ≤ 1 mm when compared with Breslow > 1 mm, with a statistically significant difference (p = 0.029); however, no difference was observed between the ISM and SSM invasive groups. This can be justified by the fact that the vast majority of cases in the invasive SSM group had Breslow ≤ 1 mm, with a mean Breslow of only 0.50 mm and the dermoscopic characteristics of thin melanomas.

Radial streaks and/or pseudopods are also more commonly found in melanomas of the SSM subtype, and it was suggested by Argenziano et al. that their presence would indicate the radial growth phase of the tumor.^38^ However, in more recent studies, these structures have been described more frequently in invasive melanomas.^15^, ^18^, ^36^

The vertical growth phase is marked by tumor invasion; a whitish-blue veil and atypical vessels are most commonly found.^41^ The veil was observed in 50% of invasive melanomas and 25% of ISM, but this difference was not statistically significant (p = 0.055), being considered a borderline finding. The veil is considered to be a highly specific structure for the diagnosis of melanoma, with a specificity of up to 97%; it is more commonly found in thicker melanomas.^42^, ^43^, ^27^, ^44^ The authors believe that this criterion could have reached statistical significance if the sample size was larger.

Milky-red areas/globules and atypical vessels appear due to vascular polymorphism, which increases proportionally to the increase in thickness, being more frequent in intermediate and thick melanomas (> 1 mm).^44^ These structures are more easily visible in hypo/amelanotic melanomas, in which they may be the only clue for diagnosis.^44^ The authors believe that the atypical vascular pattern may have been underestimated in this study, since the vessels would be better viewed with polarized light, and contact dermoscopy can compress them and impair their evaluation in the images.^45^

An inverted network is characteristic of but not exclusive to melanomas, and can be seen in Spitz's nevus, atypical nevus, or dermatofibroma.^24^, ^46^ In the present study, it was observed in 17.2% of cases, most frequently in the SSM group with Breslow > 1 mm (p = 0.018), a finding that is in accordance with the literature.^47^, ^48^ The inverted network was found more frequently in invasive melanomas with Breslow > 1 mm associated with nevi, those located in the trunk, and in young patients.^48^ Its extent and distribution were also assessed, and its presence in more than 20% of the lesion and with heterogeneous distribution were more common in invasive melanomas.^48^

The dermoscopic island, described by Borsari et al., consists of a circumscribed area with a different dermoscopic pattern from the rest of the lesion, and was considered characteristic of thin melanomas arising from nevi.^26^ In this study, it was found in only three cases (5.2%), two ISM and one invasive SSM with Breslow ≤ 1 mm, not enough to reach a statistically significant difference.

The multicomponent pattern is considered the most characteristic and most common pattern associated with melanoma; it is more common in invasive melanomas. The non-specific pattern, which was found in 10.3% of cases, was significantly more common in ISM (p = 0.028). To the best of the authors’ knowledge, this finding had not been previously described in the literature, but it is expected, since as the tumor thickness increases, more specific dermoscopic criteria for melanoma are found. It can be inferred that further studies are needed on featureless melanomas in order to better establish their diagnostic criteria. The existence of non-specific melanomas reinforces the importance of the clinical history, and biopsy should be considered for all pigmented lesions in which a benign diagnosis cannot be made, especially in the case of a lesion of recent onset and/or presenting changes and symptoms.

Dermoscopic evaluation is of fundamental importance in the early diagnosis and in the preoperative estimate of the Breslow index of melanoma. Further studies are needed, especially in the Brazilian population, with a larger sample and a higher proportion of intermediate and thick melanomas, to confirm the findings.

## Conclusions

Melanomas in early stages presented more frequently a non-specific pattern (p = 0.028) and atypical network (p = 0.029).

Invasive melanomas most frequently presented a size ≥ 6 mm (p = 0.04), a multicomponent pattern (p = 0.026), and three or more colors (p = 0.019); in turn, intermediate and thick melanomas most often presented an inverted network (p = 0.018).

A whitish-blue veil was more common in invasive SSM, but the difference was not statistically significant. Asymmetry, atypical dots and globules, radial streaks and/or pseudopods, regression structures, hypopigmented areas, blotches, atypical vessels, milky red areas, dermoscopic islands, reticular bluish-gray areas, and non-reticular, globular, starburst, and homogeneous patterns didn’t show any significant difference among the groups studied.

## Financial support

None declared.

## Authors’ contributions

Fernanda Marques Trindade: Statistical analysis; design and planning of the study; drafting and editing of the manuscript; collection, analysis, and interpretation of data; critical review of the literature; critical review of the manuscript.

Maria Luiza Pires de Freitas: Collection, analysis, and interpretation of data; intellectual participation in propaedeutic and/or therapeutic conduct of studied cases.

Flávia Vasques Bittencourt: Approval of the final version of the manuscript; design and planning of the study; drafting and editing of the manuscript; collection, analysis, and interpretation of data; effective participation in research orientation; intellectual participation in propaedeutic and/or therapeutic conduct of studied cases; critical review of the literature; critical review of the manuscript.

## Conflicts of interest

None declared.
